# Supplementary material for: RNase H1 levels dramatically affect mitochondrial genome maintenance with little impact on nuclear R-loops in murine B cells
Source: bioRxiv. 2025 Apr 30:2025.04.30.651504. Preprint. [Version 1] doi: 10.1101/2025.04.30.651504 (PMC12190172; doi:10.1101/2025.04.30.651504)
Supplement: 1 [file NIHPP2025.04.30.651504V1-supplement-1.pdf]

bioRxiv preprint doi: https://doi.org/10.1101/2025.04.30.651504; this version posted April 30, 2025. The copyright holder for this preprint (which was not certified by peer review) is the author/funder, who has granted bioRxiv a license to display the preprint in perpetuity. It is made available under aCC-BY 4.0 International license.

Supplementary Materials

Figure S1

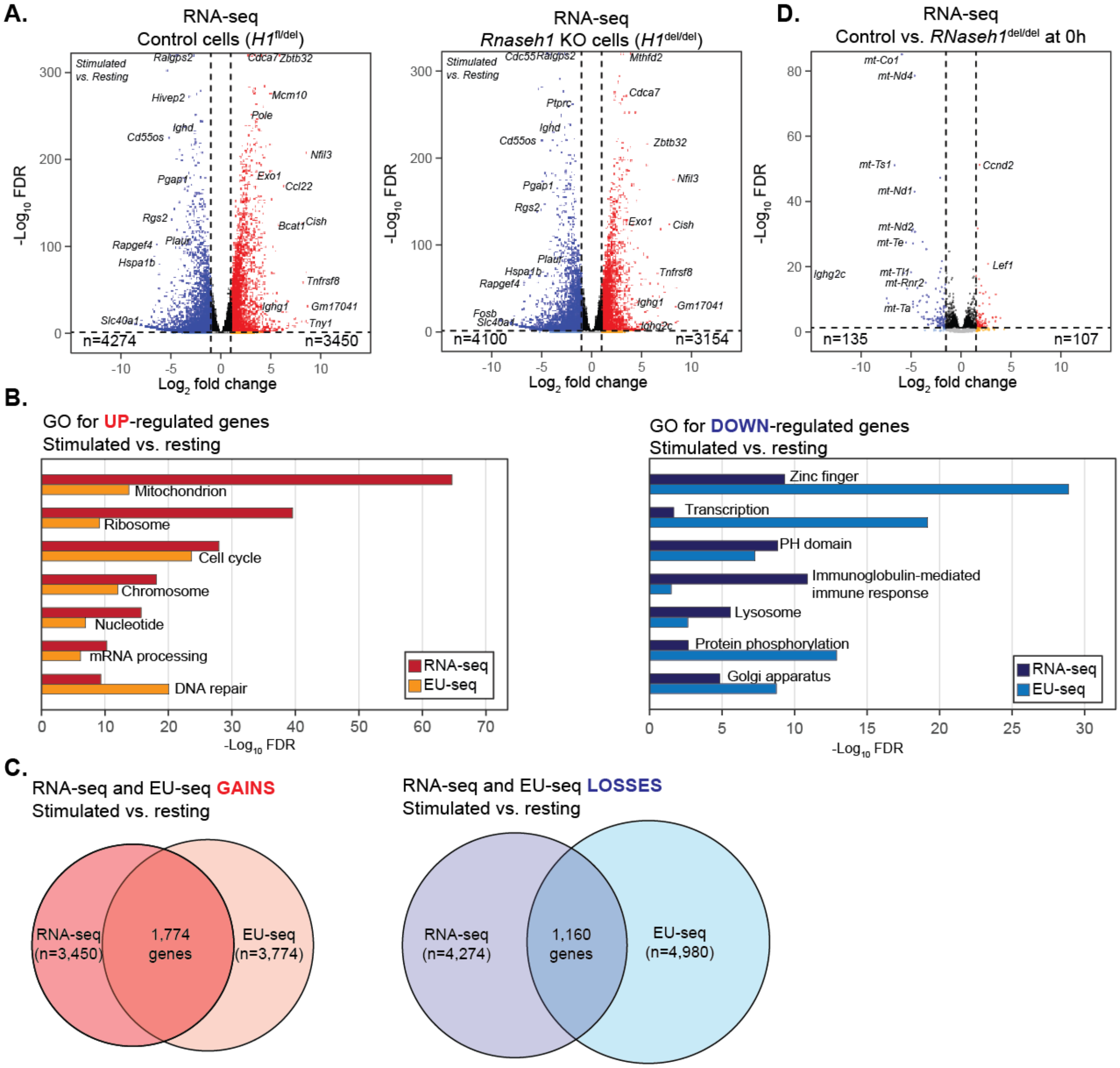

Figure S1:

A. Volcano plots of RNA-seq data comparing gene expression differences between resting and stimulated B cells in both control cells (*H1* $^{fl/del}$) and *Rnaseh1* KO cells (*H1* $^{del/del}$). Vertical dashed lines indicate $|Log_2FC|>1$ and the horizontal dashed line indicates a $Log_{10}(FDR)<0.05$. The total numbers of significantly up- or down-regulated genes are indicated.

B. Significantly enriched Gene Ontologies (David) for up- and down-regulated genes (RNA-seq and EU-seq) comparing resting and stimulated B cells in control cells (*H1* $^{fl/del}$).

bioRxiv preprint doi: https://doi.org/10.1101/2025.04.30.651504; this version posted April 30, 2025. The copyright holder for this preprint (which was not certified by peer review) is the author/funder, who has granted bioRxiv a license to display the preprint in perpetuity. It is made available under aCC-BY 4.0 International license.

C. Venn diagrams depicting overlap between RNA-seq and EU-seq data for genes that were upregulated (Gain) or downregulated (Loss) upon B cell stimulation in control cells (*H1* $^{fl/del}$).

D. Volcano plot of RNA-seq data comparing gene expression differences between control cells (*H1* $^{fl/del}$) and *Rnaseh1* KO cells (*H1* $^{del/del}$) at the resting stage.

bioRxiv preprint doi: https://doi.org/10.1101/2025.04.30.651504; this version posted April 30, 2025. The copyright holder for this preprint (which was not certified by peer review) is the author/funder, who has granted bioRxiv a license to display the preprint in perpetuity. It is made available under aCC-BY 4.0 International license.

Figure S2

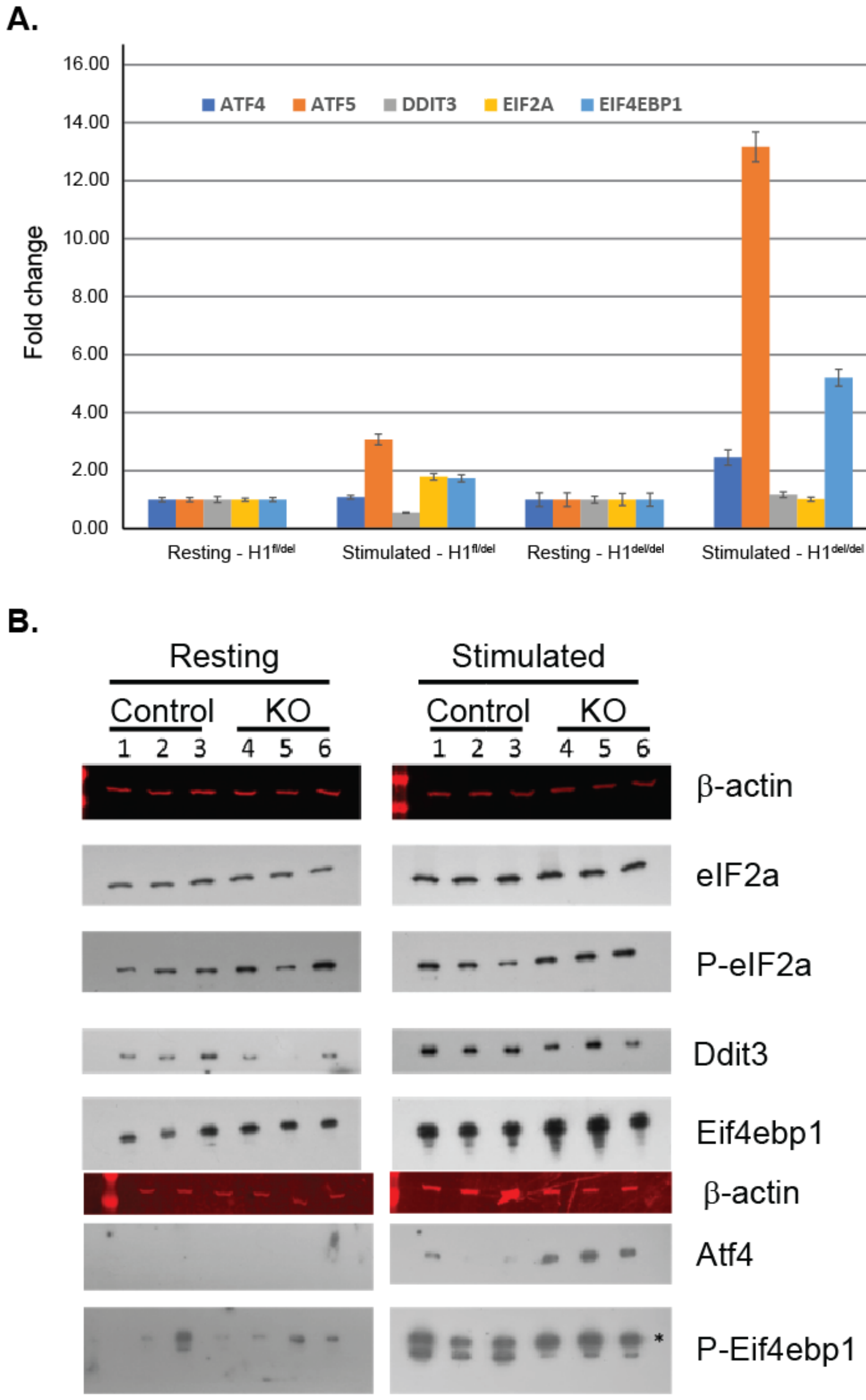

bioRxiv preprint doi: https://doi.org/10.1101/2025.04.30.651504; this version posted April 30, 2025. The copyright holder for this preprint (which was not certified by peer review) is the author/funder, who has granted bioRxiv a license to display the preprint in perpetuity. It is made available under aCC-BY 4.0 International license.

Figure S2:

A. RT-qPCR validation of the UPR^{mt} induction for key genes. Data is normalized the resting B cells. Significant induction is observed for Atf4, Atf5, and Eif4ebp1. Data is from RNA extracted from three independent animals.

B. Western blot validation for the UPR^{mt} induction. Each group displays signal from B cells extract from 3 independent animals either resting or 24 hours post-stimulation. Control group refers to $H1^{fl/del}$ mice, while KO group refers to $H1^{del/del}$ animals. The detected proteins in each panel are indicated on the right. * indicates the phosphorylated form of Eif4Ebp1. Significant increases are seen for Atf4 and P- Eif4Ebp1, with a minor increase for P-Eif2a.

bioRxiv preprint doi: https://doi.org/10.1101/2025.04.30.651504; this version posted April 30, 2025. The copyright holder for this preprint (which was not certified by peer review) is the author/funder, who has granted bioRxiv a license to display the preprint in perpetuity. It is made available under aCC-BY 4.0 International license.

Figure S3

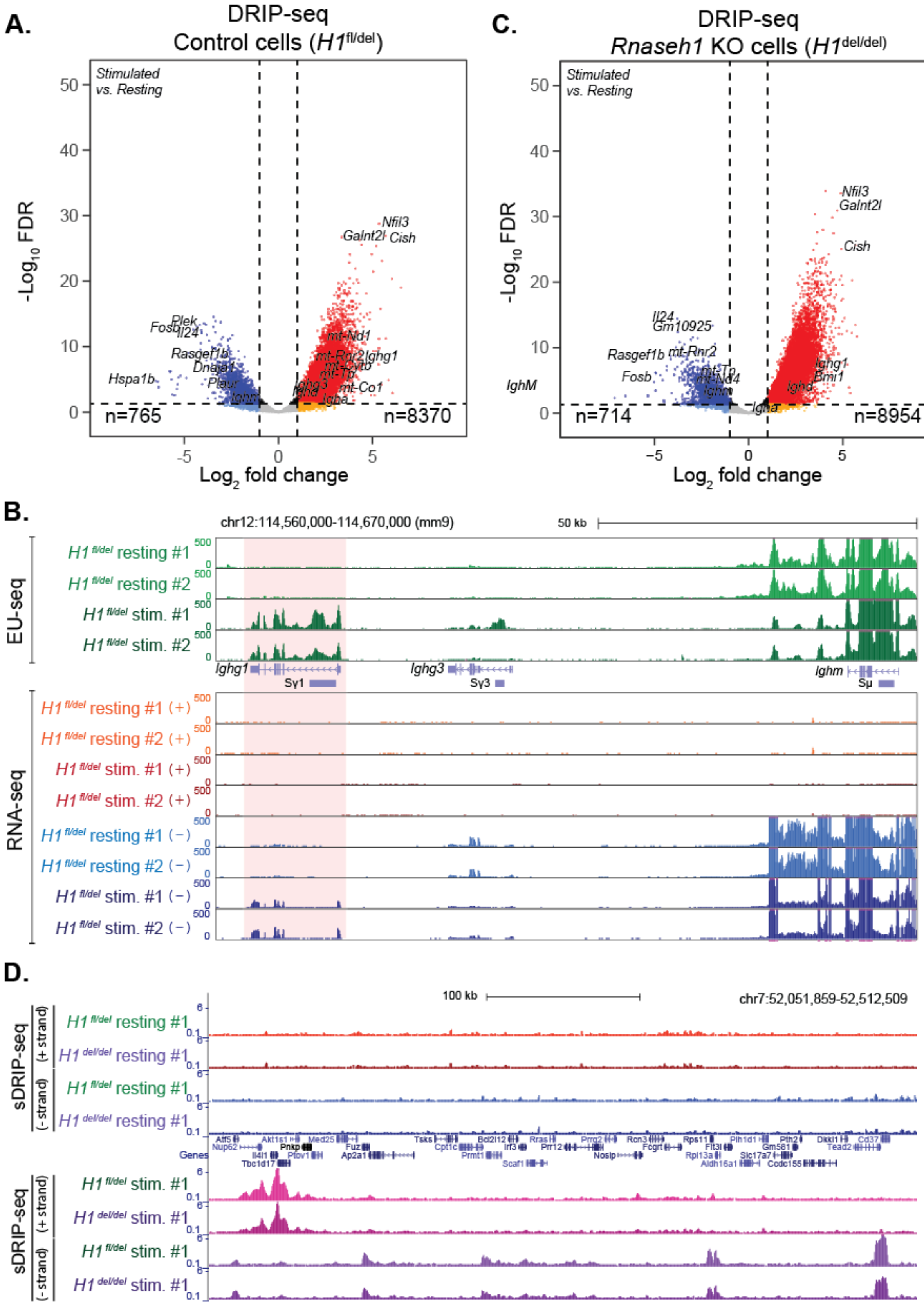

bioRxiv preprint doi: https://doi.org/10.1101/2025.04.30.651504; this version posted April 30, 2025. The copyright holder for this preprint (which was not certified by peer review) is the author/funder, who has granted bioRxiv a license to display the preprint in perpetuity. It is made available under aCC-BY 4.0 International license.

Figure S3:

A. Volcano plots of DRIP-seq data comparing R-loop intensities between resting and stimulated cells for control (*H1* $^{fl/del}$) cells. Vertical dashed lines indicate $|Log_2FC|>1$ and the horizontal dashed line indicates a $Log_{10}(FDR)<0.05$. The number of loci that are significantly up- or down-regulated are indicated.

B. Genome browser screenshot showing nascent transcription (EU-seq, top) and RNA-seq (bottom) over the IgH region for control samples at the resting and stimulated stages, as indicated. The IgG1 region is highlighted.

C. As with panel A but for resting and stimulated *H1*$^{del/del}$ B cells.

D. Genome browser screenshot over a representative autosomal region depicting strand-specific sDRIP-seq data in both control and KO cells at the resting (top) and stimulated (bottom) stages. Both cell types show similar R-loop induction patterns.

bioRxiv preprint doi: https://doi.org/10.1101/2025.04.30.651504; this version posted April 30, 2025. The copyright holder for this preprint (which was not certified by peer review) is the author/funder, who has granted bioRxiv a license to display the preprint in perpetuity. It is made available under aCC-BY 4.0 International license.

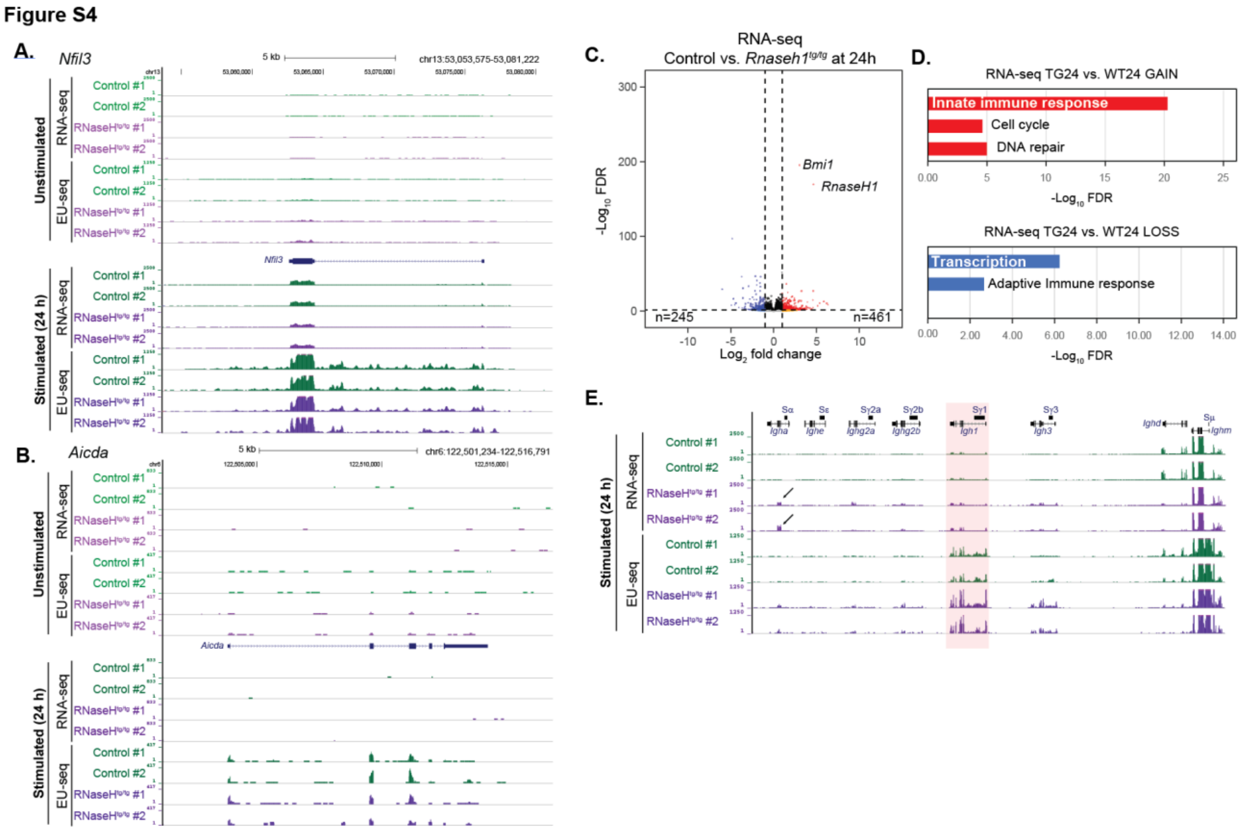

Figure S4.

Genome browser screenshots showing gene expression data (RNA-seq and EU-seq) for the *Nfil3* (**A**) and *Aicda* genes (**B**) in both control and RNase H1-overexpressing cells.

C. Volcano plots of RNA-seq data comparing gene expression levels between control and RNase H1-overexpressing cells 24 hours post-stimulation.

D. Enriched GOs observed when comparing gene expression patterns (RNA-seq) between control and RNase H1-overexpressing cells 24 hours post-stimulation.

E. Genome browser screenshot showing gene expression data (RNA-seq and EU-seq) over the IgH region for control and RNase H1-overexpressing cells 24 hours post-stimulation.

bioRxiv preprint doi: https://doi.org/10.1101/2025.04.30.651504; this version posted April 30, 2025. The copyright holder for this preprint (which was not certified by peer review) is the author/funder, who has granted bioRxiv a license to display the preprint in perpetuity. It is made available under aCC-BY 4.0 International license.

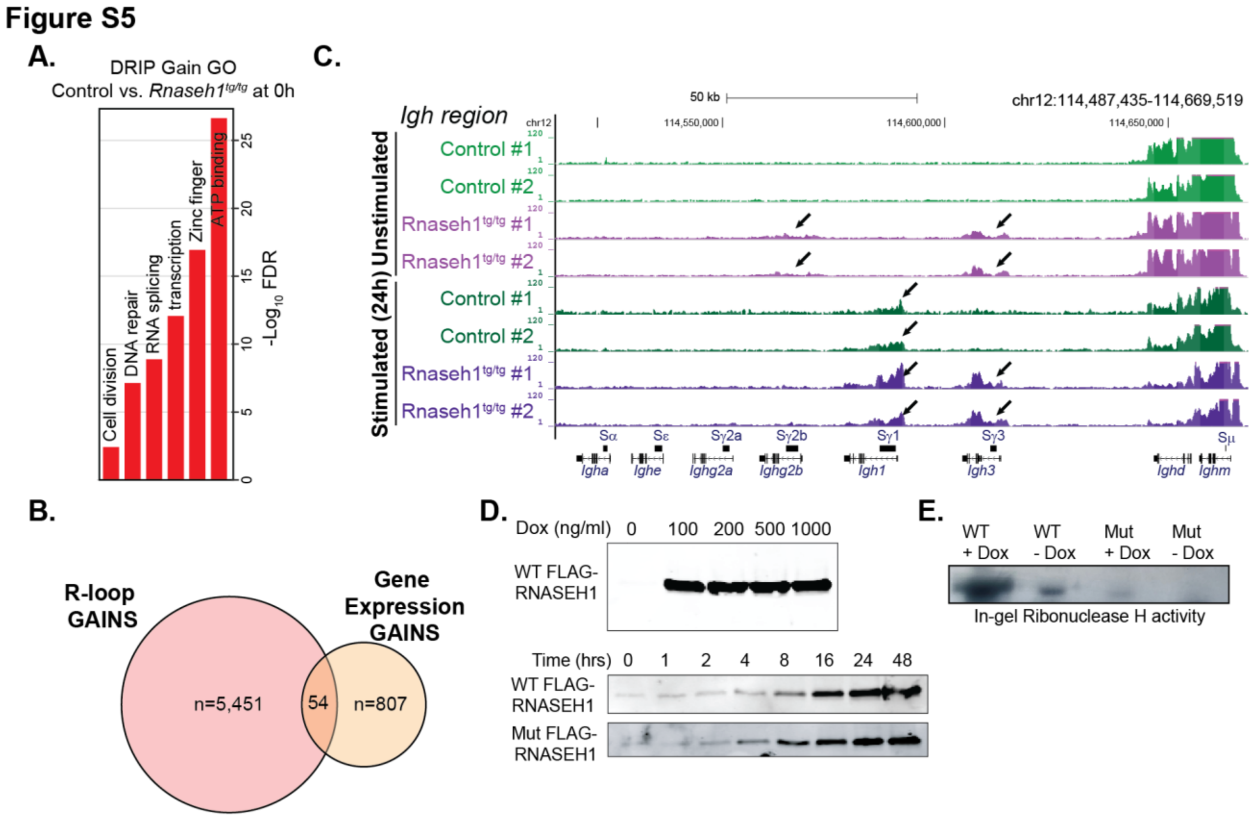

Figure S5.

A. Enriched GOs observed when comparing R-loop data (DRIP-seq) between resting control and RNase H1-overexpressing cells.

B. Venn diagrams depicting overlap between genes showing R-loop gains and gene expression gains when comparing resting control and RNase H1-overexpressing cells.

C. Genome browser screenshot showing R-loop distribution over the IgH region for control and RNase H1-overexpressing cells at both the resting and stimulated stages.

D. (top) Western blot showing induction of inducible nuclear RNase H1 expression in HEK293T cells as a function of doxycycline (Dox) addition; samples were collected 24 hours post induction. (bottom) Western blot showing time-dependent induction of RNase H1 expression in HEK293T cells post Dox induction (100 ng/ml).

E. Representative RNase H1 gel renaturation assay showing RNase H1 activity against labeled RNA/DNA hybrids in lysates generated from an equal number of HEK293T cells expressing, or not, the wild-type or mutant RNase H1 protein.
